# Overexpressing STAMP2 attenuates adipose tissue angiogenesis and insulin resistance in diabetic ApoE^−/−^/LDLR ^−/−^ mouse *via* a PPARγ/CD36 pathway

**DOI:** 10.1111/jcmm.13233

**Published:** 2017-06-19

**Authors:** Feng Wang, Lu Han, Ran‐ran Qin, Yao‐yuan Zhang, Di Wang, Zhi‐Hao Wang, Meng‐Xiong Tang, Yun Zhang, Ming Zhong, Wei Zhang

**Affiliations:** ^1^ The Key Laboratory of Cardiovascular Remodeling and Function Research Chinese Ministry of Education and Chinese Ministry of Health The State and Shandong Province Joint Key Laboratory of Translational Cardiovascular Medicine Department of Cardiology Qilu Hospital of Shandong University Jinan Shandong China; ^2^ Department of General Practice Qilu Hospital of Shandong University Jinan Shandong China; ^3^ Department of Geriatric Medicines Qilu Hospital of Shandong University Jinan Shandong China; ^4^ Department of Emergency Qilu Hospital of Shandong University Jinan Shandong China

**Keywords:** STAMP2, PPARγ, CD36, adipose tissue, angiogenesis, insulin resistance

## Abstract

The aim of this study was to investigate whether overexpression of STAMP2 improves insulin resistance by regulating angiogenesis in adipose tissues. The characteristics of diabetic mice were measured by serial metabolite and pathology tests. Samples were obtained from epididymal, subcutaneous and brown adipose tissues. Histological and morphological analysis demonstrated that STAMP2 gene overexpression reduced adipocyte size, angiogenesis in epididymal and brown adipose tissues. On aortic ring assay, microvessels sprouting from aortas were significantly inhibited after STAMP2 gene overexpression. The cellular effect of STAMP2 on angiogenesis was explored in human umbilical vein endothelial cells (HUVECs) model. Correlation of STAMP2 and angiogenesis was validated by Ad‐STAMP2 transfection and STAMP2 siRNA inhibition. *In vitro*, overexpression of STAMP2 significantly inhibited endothelial cell migration, tube formation. The effects of Ad‐STAMP2 transfection on HUVECs were abolished by treatment with PPARγ antagonist GW9662 (2.5 μM), and the roles of STAMP2 siRNA on HUVECs were also reversed by treatment with PPARγ agonist rosiglitazone (RSG) (0.1 mM). RT‐PCR indicated that STAMP2 could regulate levels of adhesion molecules, vascular endothelial growth factor A and CD36. The expression of PPARγ and CD36 was decreased when STAMP2 was inhibited by siRNA, while PPARγ and CD36 were highly expressed after overexpression of STAMP2. Our results suggested that STAMP2 gene overexpression may improve insulin resistance *via* attenuating angiogenesis in epididymal and brown adipose tissues through the PPARγ/CD36 signalling pathway.

## Introduction

Adipose tissue is considered to be one of the main driving forces for the development of insulin resistance (IR) 1. The vasculature as a key anatomical structure to furnish nutrition to adipose tissues plays a vital role in the pathogenesis of IR. It has been demonstrated that angiogenesis could promote or improve IR *via* regulating the adipogenesis and energy balance 2, 3. Adipose tissues consist of white and brown adipose tissues (WAT and BAT). Previous studies only focused on the role of WAT or BAT angiogenesis in IR respectively 4, 5, 6. The role of different adipose tissue angiogenesis in IR remains unclear.

Adipose tissue angiogenesis is closely related to IR 3, 7, 8. As reported in obese patients, hypertrophic adipocytes in WAT can increase proangiogenic factors secretion and promote angiogenesis which in turn promote adipocytes hypertrophy 9, 10, thereby creating a vicious circle and aggravating IR. In the metabolically active BAT, angiogenesis can increase energy consumption and improve IR 11.

PPARγ is present in endothelial cells and multiple studies demonstrated that PPARγ activation suppressed the proliferation of endothelial cells *in vitro* and their differentiation into tube‐like structures 12, 13, 14, 15. It has been demonstrated that PPARγ plays a major role in regulating endothelial cells apoptosis in the tumour tissue *via* CD36 16, 17. Six transmembrane protein of prostate 2 (STAMP2) is well known for its anti‐inflammatory and metabolic role in maintaining normal glucose tolerance, lipid metabolism and improving IR 18, 19, 20. Recent studies have shown that STAMP2 is a metalloreductase which may regulate cellular iron in cancer growth, angiogenesis and metastasis 21, 22. However, the effects of STAMP2, particularly in adipose tissues, on angiogenesis‐related development of insulin resistance and the underlying mechanisms are still unknown. STAMP2 knockdown leads to inhibition of adipogenesis by diminishing the expression of PPARγ 23. However, whether STAMP2 regulate the angiogenesis of adipose tissue through the PPARγ/CD36 signalling pathway remains to be further investigated.

With the aim of evaluating the effects of STAMP2 on adipose tissue angiogenesis in different adipose tissue of diabetic mouse, we established the type 2 diabetes ApoE^−/−^/LDLR^−/−^ mice models with STAMP2 gene overexpression *in vivo*. We studied the effects of STAMP2 on angiogenesis in different regional adipose tissues and determined the molecular mechanism whereby STAMP2 modulates PPARγ/CD36 signalling pathways in human umbilical vein endothelial cells.

## Materials and methods

### Diabetic model and *in vivo* experiments

Three‐week‐old male ApoE^−/−^/LDLR^−/−^ mice were fed a high‐fat diet (34.5% fat, 17.5% protein, 48% carbohydrate; Beijing HFK Bio‐Technology, China). After 6 weeks, the ApoE^−/−^/LDLR^−/−^ mice underwent an intraperitoneal glucose tolerance test (IPGTT). Those mice showing IR were injected once with low dose of streptozotocin (STZ, Sigma‐Aldrich, St. Louis, MO, USA; 75–80 mg/kg i.p. in 0.1 mol/l citrate buffer, pH 4.5) intraperitoneally. After 2 weeks, most high‐fat diet/STZ‐treated mice displayed hyperglycemia, IR and glucose intolerance, as previously reported 24. At age 11 weeks, mice with similar degrees of hyperglycemia and body weight were randomly divided into two groups, one will be treated with the STAMP2‐expressing adenoviruses, referred to as the DM + STAMP2 group (*n* = 10), and the other treated with the expressing vector control adenovirus, referred to as the DM + vehicle (*n* = 10; see later for the details of the adenovirus used). The mice fed a normal diet were used as non‐diabetic controls, divided into control + vehicle (*n* = 10) and control + STAMP2 (*n* = 10) groups. All animal procedures were performed in accordance with animal protocols approved by Shandong University Institutional Animal Care and Use Committee.

### Intraperitoneal glucose tolerance test (IPGTT)

Glucose tolerance was assessed by IPGTT after mice fasted for 12–16 hrs. A bolus of glucose (2 g/kg) was injected intraperitoneally, and blood samples were collected from the tail vein at 0, 15, 30, 60 and 120 min and glucose was measured using a One touch Glucometer (Life‐Scan, Milpitas, CA, USA).

### Production and administration of adenoviral vector

The cDNA of mouse STAMP2 (GenBank accession no. BC006651) from SinoGenoMax Company Limited was cloned into the p‐Shuttle vector. STAMP2 cDNA was subcloned between KpnI and EcoRI of the p‐Shuttle expression cassette. Then recombinant pAdxsi adenovirus was constructed using the pAdxsi Adenoviral System (SinoGenoMax, Beijing, China). After amplification, viruses were purified, tittered and stored at −80°C until used. All mice were injected *via* the jugular vein with 5 × 10^9^ plaque‐forming units of virus at 20 weeks. Adenovirus transfer was repeated at 22 weeks. The control group was injected with control virus (vehicle). Four weeks after first adenovirus injection, all mice were killed for further study.

The recombinant pENTER adenovirus constitutively expressing human STAMP2 was constructed using the pENTER Adenoviral System (no. CH835357 Vigene, Jinan, China). Then STAMP2 cDNAs from human were inserted into pENTER‐CMV vector. The pENTER‐EGFP vector adenovirus was used as the control vehicle virus. The recombinant pENTER adenovirus was use for cell transfection.

### Blood analyses

At the end of all experiment, we collected the murine samples to measure the levels of fasting blood glucose, total cholesterol, total triglyceride and free fatty acids.

### Histological and morphometric analyses

Samples were taken from epididymal, subcutaneous and brown adipose tissue (EWAT, SWAT and BAT). Each adipose tissue sample was cut into two pieces. One‐half of the samples were fixed in paraformaldehyde (4%) and embedded in paraffin, and cut into 5 mm sections. A single adipocyte was measured with images captured from haematoxylin and eosin‐stained sections. Every adipocyte area was assessed under ×400 magnification within adipose tissue, and a mean was obtained by quantitative morphometry with automated image analysis (Image‐Pro Plus, Version 5.0; Media Cybernatics, Houston, TX, USA).

### *Ex vivo* aortic ring angiogenesis assay

Aortas of mice were dissected for aortic ring assay as described with some modifications 25. Briefly, dissected thoracic aortas from treated ApoE^−/−^/LDLR^−/−^ mice were cut into about 0.5 mm long rings. Some rings were transfected with 1 × 10^6^ PFU/ml STAMP2‐overexpressing adenovirus overnight. Then all aortic rings were embedded in matrigel (Cat. No. 356234, BD, USA). After the matrix polymerize, aortic rings were incubated with 2.5% foetal bovine serum (FBS) Opti‐MEM and medium was changed on days 3, 6 and 8. The VEGF treatment was supplemented to a final concentration of 30 ng/ml. Pictures were captured by use of a Canon Camera linked to a light microscope on day 8 and the number of microvessels sprouting was counted.

### Immunohistochemical staining

Paraffin sections underwent immunohistochemistry by a microwave‐based antigen retrieval method. The sections were incubated with antimouse endomucin (1:200 eBioscience Inc., California, USA) and CD31 (1:100 ab28364) overnight and then with a matching biotinylated secondary antibody for 30 min at 37°C. Negative controls were omission of the primary antibody. The stained sections were developed with diaminobenzidine and counterstained with haematoxylin. The results were viewed under a confocal FV 1000 SPD laser scanning microscope (Olympus, Japan).

### Cell culture

Primary human umbilical vein endothelial cells (HUVECs) were purchased from American Type Culture Collection. HUVECs were grown in ECM medium (Sciencell, 6076 Corte Del Cedro Carlsbad, USA) supplemented with 10% FBS, 100 U/ml penicillin, 100 μg/ml streptomycin. Human umbilical vein endothelial cells within five passages were used in the following experiments.

### SiRNA transfection

Transfection was performed with Lipofectamine 2000 reagent (5 μl, per well/6‐well plate, Invitrogen). Cells were transfected with 100 pmol siRNA (siRNA‐STAMP2, 5 μl, per well/6‐well plate) or 100 pmol negative control siRNA (siRNA‐NC, 5 μl, per well/6‐well plate). Transfection was performed using the following primers: STAMP2: forward 5′‐UGCAGAGUACCUUGCUCAUTT‐3′ and reverse 5′‐AUGAGCAAGGUACUCUGCATT‐3′; Negative control: forward 5′‐UUCUCCGAACGUGUCACGUTT‐3′ and reverse 5′‐ACGUGACACGUUCGGAGAATT‐3′. After incubation for 6 hr, culture medium should be changed. Then the cells were observed under a laser scanning confocal microscopy (LeicaTCSSP2; Leica).

### Transfection of STAMP2 overexpressing adenovirus

The cells were administered virus in 200 MOI. The culture medium was changed after 12 hrs. Then the cells were observed under a laser scanning confocal microscopy (LeicaTCSSP2; Leica).

### Cell migration assay

Migration of cells was examined using the wound assay as previously described 26. HUVECs were treated with siRNA‐NC, siRNA‐STAMP2, siRNA‐STAMP2 + RSG (0.1 mM, pretreated 1 hr, ab120762), Ad‐NC, Ad‐STAMP2 or Ad‐STAMP2 + GW9662 (2.5 μM, pretreated 1 hr, Sigma‐M6191), respectively. After 48 hrs, confluent cell monolayers were wounded by use of a yellow tip. Detached cells were washed away and then fresh low serum medium was added. After 12 hrs of incubation, cells that had migrated across the edge of the wound and into the gap were counted as migrating cells. Images were photographed at 0 and 12 hrs after scratching. The closure of the wounded area was analysed by use of Image‐pro plus 6.0.

### Transwell migration assay

HUVECs from different groups (siRNA‐NC, siRNA‐STAMP2, siRNA‐STAMP2 + RSG, Ad‐NC, Ad‐STAMP2 or Ad‐STAMP2 + GW9662) were seeded in the upper chamber, and the lower chambers were filled with low serum ECM medium. Cells were allowed to migrate for 4 hr. Non‐migrated cells were removed and migrated cells on the lower side of the membrane were stained with crystal violet. Images were captured in five random fields (×100).

### Tube formation assay

Tube formation assay involved use of growth factor reduced Matrigel (Cat. No. 356234, BD, USA). Briefly, after matrigel matrix gelled, HUVECs from different groups (siRNA‐NC, siRNA‐STAMP2, siRNA‐STAMP2 + RSG, Ad‐NC, Ad‐STAMP2 or Ad‐STAMP2 + GW9662) (1 × 10^5^ cells per well/48‐well plate) were suspended in low serum medium. The formation of capillary‐like tubes was captured 12 hrs later. The mean tube length was calculated in five random fields (×100).

### Quantitative real‐time RT‐PCR

Total RNA samples were prepared from HUVECs transfected with siRNA‐NC, siRNA‐STAMP2, siRNA‐STAMP2 + RSG, Ad‐NC, Ad‐STAMP2 or Ad‐STAMP2 + GW9662, respectively. First strand cDNA was generated using the first‐strand cDNA synthesis kit for reverse transcription‐polymerase chain reaction (RR037A Takara). Quantitative RT‐PCR was performed with a LightCycler system (Roche Diagnostics) according to the manufacturer's instructions. Polymerase chain reaction use Ultra SYBR (CW0957s cwbiotech). The sense and antisense primer pairs used were as follows: STAMP2, Forward 5′‐ATGACAGCAAAGCCAAGCAA‐3′, Reverse 5′‐GCAAAGCATCCAGTGGTCAA‐3′; VCAM‐1, Forward 5′‐ATGACCTTCATCCCTACCATTGA‐3′, Reverse 5′‐CATTGACATAAAGTGTTTGCGTACTCT‐3′; ICAM‐1, Forward 5′‐ACGTACCTCTATAACCGCCAGC‐3′, Reverse 5′‐ATATGGGAAGGCCGAGGAAGAG‐3′; VEGF‐a, Forward 5′‐CGGCGAAGAGAAGAGACACA‐3′, Reverse 5′‐GGAGGAAGGTCAACCACTCA‐3′; Flt‐1, Forward 5′‐ACCCAGATGAAGTTCCTTTGGA‐3′, Reverse 5′‐CCCAGTTTAGTCTCTCCCGG‐3′; CD36, Forward 5′‐ATGGGCTGTGACCGGAACT‐3′, Reverse 5′‐ACAGACCAACTGTGGTAG‐3′; GAPDH, Forward 5′‐GTCAGCCGCATCTTCTTTTG‐3′, Reverse 5′‐GCGCCCAATACGACCAAATC‐3′.

### Western blot analysis

Western blot analysis was as previously described 27. We used antibodies against STAMP2 (1:1000 Cat. No. 11944‐1‐AP Protein‐tech Group Inc., Chicago, IL, USA), PPARγ (1:1000 ab191407), CD36 (1:1000 ab133625), followed by anti‐IgG horseradish peroxidase‐conjugated secondary antibody. STAMP2, PPARγ, CD36 protein level was normalized to that of β‐actin (1:1000 Cat. No. ZM0003 ZSGB‐bio Beijing) or GAPDH (1:1000 Cat. No. TA336621, ZSGB‐bio Group Inc., Beijing, China) as an internal control.

### Statistical analysis

Values are presented as mean ± SD. SPSS 17.0 (SPSS, Chicago, IL, USA) was used for statistical analysis. Results were compared by one‐way ANOVA, followed by Tukey–Kramer post hoc test and independent samples *t*‐test. *P *< 0.05 was considered statistically significant.

## Results

### Overexpression of STAMP2 improves metabolism in ApoE^−/−^LDLR^−/−^ mice

Consistent with our previous publication 28, diabetes mellitus (DM) induced by high fat and sugar diet combined with a small dose of STZ in male ApoE^−/−^/LDLr^−/−^ mice could resemble human diabetes mellitus and STAMP2 overexpression decreased risk factor of insulin resistance in diabetic ApoE^−/−^LDLR^−/−^ mice (Fig. S1, Table S1).

### WAT and BAT morphology

This result was consistent with the our former report 28, STAMP2 overexpression reduced adipocyte size in epididymal white adipose tissue (EWAT) and lipid area in brown adipose tissues (BAT). However, overexpression of STAMP2 has no effect on adipocyte size in SWAT (Fig. S2) .

### STAMP2 gene overexpression inhibits angiogenesis *ex vivo*

**Figure 1:**
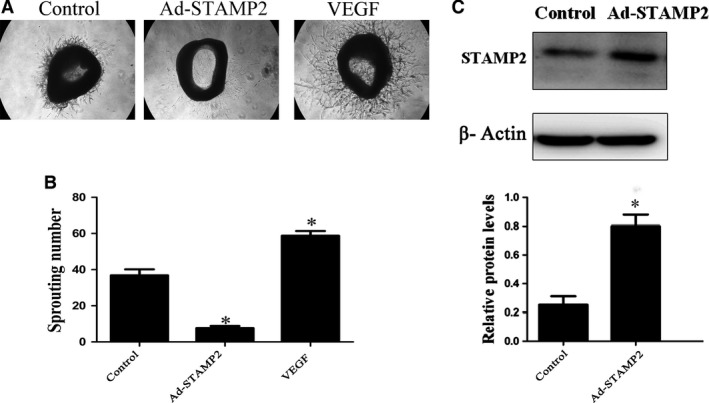
STAMP2 gene overexpression inhibits angiogenesis *ex vivo*. (**A**) Representative images of mouse aortic ring sprouts with three treatment groups. (**B**) Quantification of sprouting number in aortic rings. (**C**) Quantification of STAMP2 overexpression in aortic tissue at the protein level. Data are mean ± SD. *P < 0.05 *versus* control.

We observed the effect of STAMP2 overexpression on angiogenesis *ex vivo* by mouse aortic ring assay. The numbers of sprouting microvessel branches from aortic rings were reduced with STAMP2 overexpression than control treatment (*P *< 0.05) (Fig. 1).

### STAMP2 gene overexpression suppresses angiogenesis in EWAT and BAT

**Figure 2:**
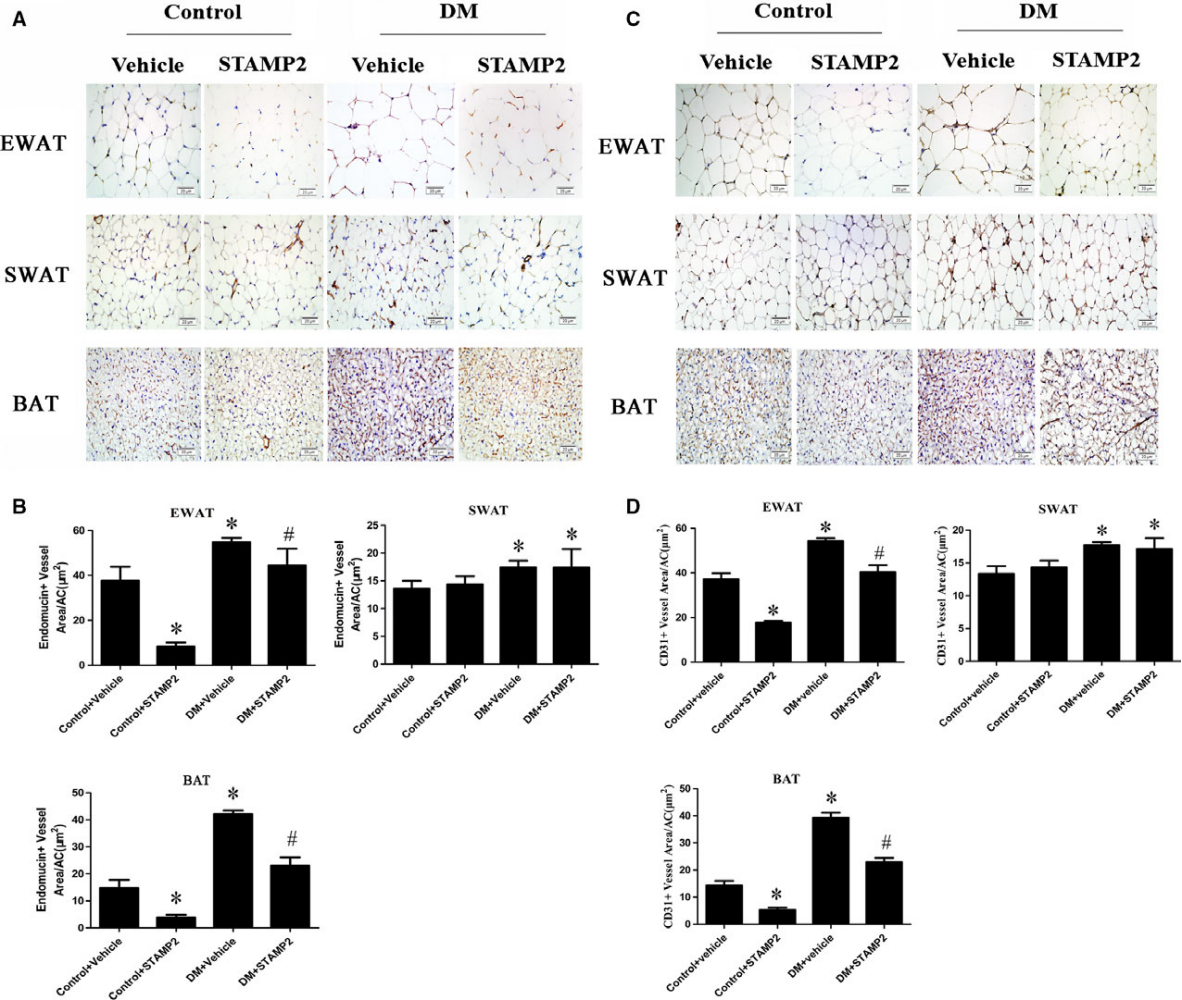
STAMP2 gene overexpression suppresses angiogenesis density in WAT and BAT of ApoE^−/−^/LDLR ^−/−^ mice. (**A**) Immunohistochemical staining (endomucin as angiogenesis marker) showing angiogenesis in adipose tissue (brown staining considered positive staining; scale bar: 20 μm); (**B**) Semiquantification of endomucin immunohistochemical staining. (**C**) Immunohistochemical staining (CD31 as angiogenesis marker) showing angiogenesis in adipose tissue (brown staining considered positive staining; scale bar: 20μm); (**D**) Semiquantification of CD31 immunohistochemical staining. Data are mean ± SD (*n* = 6 per group). *P < 0.05 *versus* control + vehicle; ^#^ *P* < 0.05 *versus* DM + vehicle. EWAT, epididymal white adipose tissue; SWAT, subcutaneous white adipose tissue; BAT, brown adipose tissue.

The density of angiogenesis using endomucin or CD31 as angiogenesis markers was significantly increased in WAT in DM group (*P *< 0.05; Fig. 2A and C). DM + STAMP2 group showed significantly decreased angiogenesis density in EWAT. We also found that angiogenesis density was elevated significantly in BAT in the diabetic ApoE^−/−^/LDLR^−/−^ mice group (*P* < 0.05; Fig. 2A and C). DM + STAMP2 group showed significantly decreased angiogenesis density in BAT. Moreover, we found that angiogenesis density in EAT and BAT of control + STAMP2 group were much less than that of control + vehicle (*P* < 0.05; Fig. 2A and C). Those changes above were not seen in SWAT in DM + STAMP2 group compared to DM + vehicle. These data (Fig. 2B and D) suggested that STAMP2 overexpression can suppress angiogenesis in EWAT and BAT.

### STAMP2 gene overexpression increases expression level of PPARγ/CD36 in EWAT and BAT

**Figure 3:**
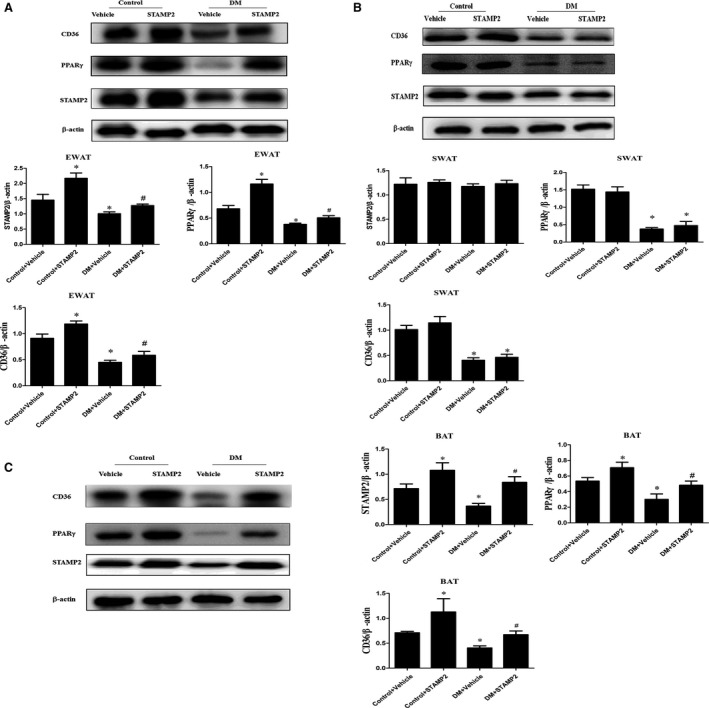
Western blot analyses of STAMP2, PPARγ and CD36 in WAT and BAT from ApoE^−/−^/LDLR ^−/−^ mice adipose tissues (*n* = 3 mice per group). The Western blots are representative of three separate experiments that gave similar results. (**A**) Representative Western blot of EWAT; (**B**) Representative Western blot of SWAT; (**C**) Representative Western blot of BAT. Data are mean ± SD; *P < 0.05 *versus* control + vehicle; ^#^ *P* < 0.05 *versus* DM + vehicle. EWAT, epididymal white adipose tissue; SWAT, subcutaneous white adipose tissue; BAT, brown adipose tissue.

Consistent with our previous publication 28, endogenous STAMP2 expression was significantly decreased in EWAT and BAT in DM + vehicle group compared with that in control + vehicle group (*P* < 0.05) (Fig. 3A and C). Overexpression of STAMP2 could significantly increase STAMP2 expression in EWAT and BAT in diabetic ApoE^−/−^/LDLR^−/−^ mice (*P* < 0.05) (Fig. 3A and C). However, there were no significant differences in STAMP2 expression in subcutaneous white adipose tissues (SWAT) in all groups. With STAMP2 overexpression treatment, the protein expression level of PPARγ/CD36 was marked increased in EWAT and BAT (*P* < 0.05) (Fig. 3A and C). However, the changes above in protein expression level of PPARγ/CD36 were not seen in SWAT in control + STAMP2 and DM + STAMP2 (Fig. 3B).

### The effect of STAMP2 gene on HUVECs migration and tube formation *in vitro*

In this study, we examined the function of STAMP2 *in vitro*. We treated HUVECs with siRNA‐NC, siRNA‐STAMP2, siRNA‐STAMP2 + RSG, Ad‐NC, Ad‐STAMP2 or Ad‐STAMP2 + GW9662 and examined their effects on angiogenesis‐related properties of HUVECs.

**Figure 4:**
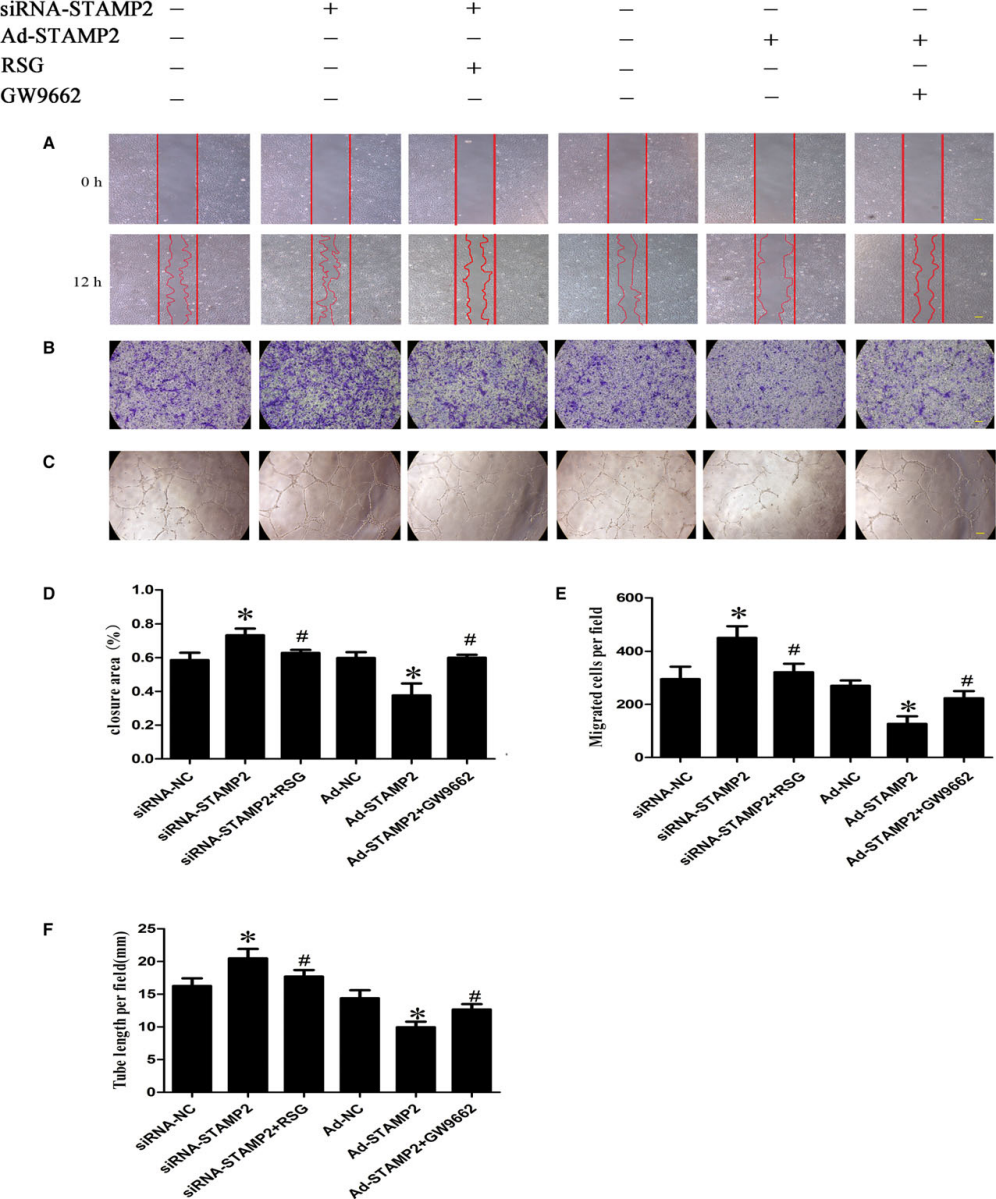
Effect of STAMP2 on HUVECs migration and tube formation *in vitro* (*n* = 3 per group). (**A**) Representative images of wound‐healing assay at 0 and 12 hrs, respectively, showing that STAMP2 silencing increased human umbilical vein endothelial cell migration, whereas STAMP2 overexpression inhibited human umbilical vein endothelial cell migration. After activation or inhibition PPARγ by RSG or GW9662, the effects of STAMP2 silencing or STAMP2 overexpression on HUVECs are reversed. (scale bar: 100 μm); (**B**) Quantitative analysis of wound‐healing assay in six groups of human umbilical vein endothelial cells at 12 hrs. (**C**) Representative images of Transwell assay showing that STAMP2 silencing increased human umbilical vein endothelial cell migration, whereas STAMP2 overexpression inhibited human umbilical vein endothelial cell migration. After activation or inhibition PPARγ by RSG or GW9662, the effects of STAMP2 silencing or STAMP2 overexpression on HUVECs are reversed. (scale bar: 100 μm); (**D**) Quantitative analysis of transwell assay in six groups of human umbilical vein endothelial cells. (**E**) Representative images of tube formation assay showing STAMP2 silencing increased human umbilical vein endothelial tube formation, whereas STAMP2 overexpression inhibited human umbilical vein endothelial tube formation. After activation or inhibition PPARγ by RSG or GW9662, the effects of STAMP2 silencing or STAMP2 overexpression on HUVECs are reversed. (scale bar: 100 μm). (**F**) Quantitative analysis of tube formation assay in six groups of human umbilical vein endothelial cells. Data are mean ± SD; *P < 0.05 *versus* siRNA‐NC group/Ad‐NC group; ^#^ *P* < 0.05 *versus* siRNA‐STAMP2 group/Ad‐STAMP2 group.

The migration of endothelial cells to designated sites is a critical step in angiogenesis 29. Thus, we used wound‐healing assay to observe the effects of STAMP2 on cell motility. STAMP2 silencing in the siRNA‐STAMP2 group significantly promoted wound closure as compared with the siRNA‐NC group, which was completely abolished by RSG (*P* < 0.05) (Fig. 4A and B). As expected, cell from Ad‐STAMP2 group showed significantly lower migration ability compared with from Ad‐NC group, which was antagonized by GW9662 (*P* < 0.05) (Fig. 4A and B). This effect of STAMP2 on HUVECs migration was further confirmed by transwell assay (*P* < 0.05) (Fig. 4C and D).

HUVECs spontaneously form tubes when plated on Matrigel. Whereas siRNA‐NC group formed tube structures on Matrigel surfaces, this tube‐forming ability was heightened in HUVECs treated with siRNA‐STAMP2 (*P* < 0.05) (Fig. 4E and F). As expected, tube formation was reduced in Ad‐STAMP2 compared with Ad‐NC group. The effects of Ad‐STAMP2 transfection on tube formation were completely reversed by treatment with GW9662, while RSG significantly abolished the roles of siRNA‐STAMP2 in tube formation. These results suggested that silence or overexpression of STAMP2 affected cell abilities of migration, tube formation by PPARγ/CD36 signalling pathway.

### The role of STAMP2 in regulating the PPARγ/CD36 signalling pathway

**Figure 5:**
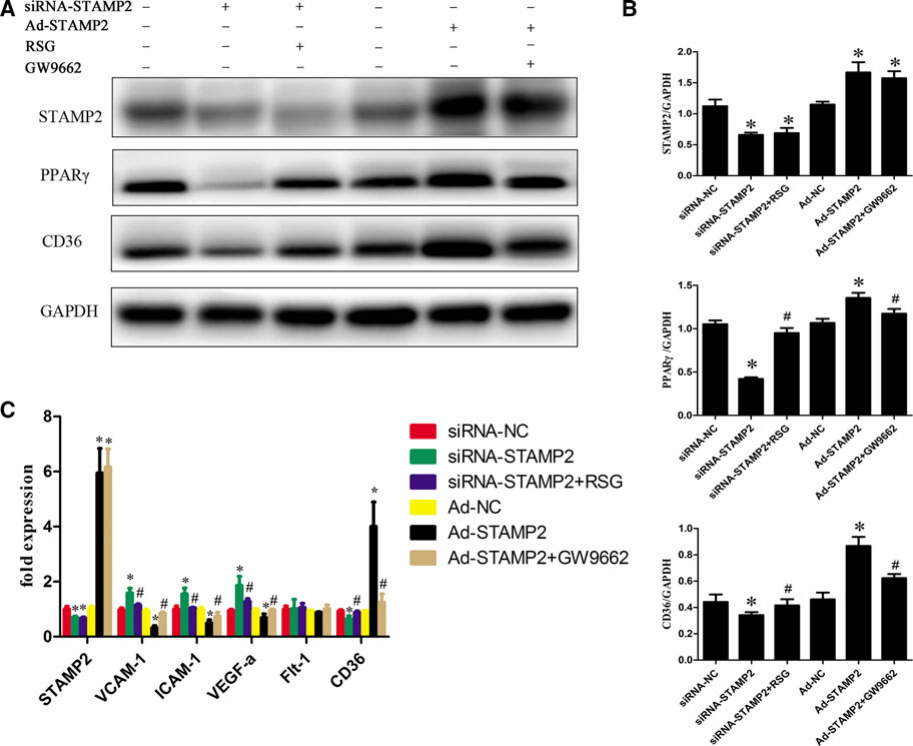
Role of STAMP2 in regulating the PPARγ/CD36 signalling Pathway and Expression of Angiogenesis‐Related Genes in HUVECs (*n* = 3 per group). (**A**) Representative images of Western blot showing that STAMP2 regulate PPARγ/CD36 signalling Pathway in six groups of human umbilical vein endothelial cells. (**B**) Western blot analysis of STAMP2, PPARγ and CD36. (**C**) Role of STAMP2 in the expression of angiogenesis‐related genes in HUVECs. Data are mean ± SD; *P < 0.05 *versus* siRNA‐NC group/Ad‐NC group; ^#^ *P* < 0.05 *versus* siRNA‐STAMP2 group/Ad‐STAMP2 group.

We investigated the role of STAMP2 in regulating the PPARγ/CD36 signalling pathway (*P* < 0.05) (Fig. 5A and B). Western blot analysis showed that the protein level of STAMP2 was lower in the siRNA‐STAMP2 group than the siRNA‐NC group (*P* < 0.05) (Fig. 5A and B). After silence of STAMP2, the protein levels of PPARγ/CD36 were reduced respectively in siRNA‐STAMP2 group compared with siRNA‐NC group. Cells were treated with the RSG significantly increased PPARγ protein level, while CD36 protein level was also highly regulated. (*P* < 0.05) (Fig. 5A and B). These data showed that STAMP2 silencing suppressed the PPARγ/CD36 signalling Pathway. As expected, the protein level of STAMP2 was increased in Ad‐STAMP2 group compared with Ad‐NC group (*P* < 0.05) (Fig. 5A and B). The protein levels of PPARγ/CD36 were increased respectively in Ad‐STAMP2 group compared to Ad‐NC group. Then we decreased PPARγ protein level using GW9662 and CD36 protein level was also downregulated. The results indicated that STAMP2 overexpressing increases the PPARγ/CD36 expression level. Taken together, these data showed that STAMP2 could regulate the PPARγ/CD36 signalling pathway in HUVECs.

### The role of STAMP2 in the expression of angiogenesis‐related genes in HUVECs

STAMP2 might alter the angiogenic properties of HUVECs by modulating the expression of angiogenesis‐related genes. To determine the role of STAMP2 in HUVECs gene expression, we compared the mRNA expression profile of siRNA‐NC group, siRNA‐STAMP2 group, siRNA‐STAMP2 + RSG, Ad‐NC group, Ad‐STAMP2 group or Ad‐STAMP2 + GW9662 (*P* < 0.05) (Fig. 5C). We found that STAMP2 overexpression could decrease VEGF‐A, ICAM‐1, VCAM‐1 and CD36 mRNA level, while it downexpression can increase VEGF‐A, ICAM‐1, VCAM‐1 and CD36 mRNA level. As expected, treatment of cells with Ad‐STAMP2 + GW9662 significantly restored VEGF‐A, ICAM‐1, VCAM‐1 and CD36 mRNA level and siRNA‐STAMP2 + RSG treatment can decrease VEGF‐A, ICAM‐1, VCAM‐1 and CD36 mRNA level compared with siRNA‐STAMP2. Taken together, these data indicated that STAMP2 can regulate angiogenesis‐related gene expression *via* PPARγ/CD36 signalling pathway.

## Discussion

In the present study, we found that overexpression of STAMP2 could effectively attenuate angiogenesis in the EWAT and BAT of diabetic ApoE^−/−^/LDLR^−/−^ mice. The molecular mechanism may involve increased expression of PPARγ/CD36 by STAMP2 overexpression. Consistent with our previous publication 28, we confirmed that overexpression of STAMP2 effectively improves metabolic indices and IR in diabetic ApoE^−/−^/LDLR^−/−^ mice, although there was no significant effect on TG level.

Relation between adipocyte size, insulin sensitivity and angiogenesis remains uncertain. Adipocyte size may be a good indicator of adipogenesis capacity; thus for a given fat mass, the more adipogenesis is active, the smaller the adipocytes are 30, 31. Insulin‐resistant state being associated with larger adipocytes 32. Adipocyte hypertrophy could directly stimulate angiogenesis 30, 33. Several reports have suggested that antiangiogenic treatment reduces body weight in murine models of obesity and lead to improvements in some aspects of the metabolic syndrome 5, 34, 35. Suppressing angiogenesis in adipose tissues induced larger adipocyte apoptosis 6. Conversely, several studies indicated that increased angiogenesis in adipose tissue results in decreased inflammation, and amelioration of HFD‐induced insulin resistance 36, 37, 38. In the present study, we demonstrated that the cell size and angiogenesis were significantly increased in adipose tissues of diabetic ApoE^−/−^/LDLR^−/−^ mice; Compared with the diabetic group, STAMP2 overexpression could significantly decrease the EWAT and BAT cell size and attenuate the angiogenesis in the EWAT and BAT of diabetic ApoE^−/−^/LDLR^−/−^ mice, with no significant change in SWAT. Moreover, it was reported that no clear differences in adipocyte size were observed between STAMP2^−/−^ mice and wild‐type mice 18, which suggested that STAMP2 was not required to maintain cell morphology. Consequently, STAMP2 gene overexpression may improve adipose tissue dysfunction through diminishing angiogenesis effectively.

The major mechanisms regulating angiogenesis in adipose tissues may involve tissue hypoxia, inflammation and oxidative stress 2, 3. However, different mechanisms may come into playing different microenvironments 39, 40, 41, 42. At early stages of obesity, hypoxia is an early determinant that increases angiogenesis in adipose tissue. However, on the stage of extremely obesity or diabetes, dysfunctional adipocytes could increase the pro‐inflammatory factors secretion, such as TNF‐α and IL‐6 that modulate an angiogenesis response in adipose tissue 43, 44 and could be a primary contributor of angiogenesis. In our previous study, we have also proved that there were a large number of inflammatory cells and inflammatory cytokines existing in adipose tissue in diabetic ApoE^−/−^/LDLR^−/−^ mice 28. It has been reported that capillary endothelial cells (ECs) influenced the transport of circulating monocytes into adipose tissue to differentiate tissue macrophages 45, 46. On the stage of extremely obesity or diabetes, oxidative stress and endoplasmic reticulum stress caused endothelial injury in adipose tissue which attract inflammatory cells such as macrophages to this site and further exacerbate the local inflammation 47, 48. Consequently, angiogenesis and inflammation are intricately associated and form a vicious cycle in promoting adipocyte dysfunction and IR. Taken together, suppressing angiogenesis could ease inflammation in adipose tissue by decreasing inflammatory cell such as macrophage infiltration into adipose tissue.

An important finding in the present study was that STAMP2 overexpression inhibited adipose tissue angiogenesis and angiogenesis‐related gene expression. PPARγ was a key regulator of angiogenesis in adipose tissues and PPARγ activation inhibited proliferation of endothelial cells *in vitro* 15, 49. In tumour xenografts, troglitazone and rosiglitazone suppressed angiogenesis and induced ECs apoptosis in a CD36‐dependent manner 16. Thus, PPARγ could be considered as a critical regulator of CD36 expression. Besides, STAMP2 knockout could inhibit adipogenesis *via* regulating PPARγ signalling pathway 23. In this study, *in vivo* and *in vitro* studies using gain‐of‐function and loss‐of‐function approaches showed that STAMP2 may regulate adipose tissue angiogenesis *via* the PPARγ/CD36 signalling pathway and the effects of STAMP2 on angiogenesis are independent of inflammation. Of course, whether STAMP2 could regulate PPARγ expression *via* superoxide production during angiogenesis would need further investigations 21, 50. In addition, STAMP2 can inhibit angiogenesis‐related gene expression in our study. STAMP2 overexpression decreased the induction of VEGF‐A, which may account for the attenuation of both adipose angiogenesis and inflammation as VEGF‐A is both a proangiogenic factor but also a recognized inflammatory marker 51, 52. The gene expression of ICAM‐1 and VCAM‐1 which interferes with monocyte adherence to endothelial cells and their subsequent migration to the subendothelial space was decreased in response to STAMP2 overexpression 53. Here, we found that STAMP2 could regulate PPARγ/CD36 signalling pathway in HUVECs cellular model, which may explain the antiangiogenic effect.

In conclusion, STAMP2 gene overexpression significantly downregulated the angiogenesis of EWAT and BAT *via* the PPARγ/CD36 signalling pathway, contributing to the improvement of IR and providing a new treatment target for diabetes and related diseases.

## Conflict of interest

The authors confirm that there are no conflicts of interest.

## Author contributions

F.W., Y.‐H.L., Z.‐H.W., Y.‐Y.Z. conceived and designed the experiments. F.W., D.W., L.H. researched data. M.‐X.T., M.Z. and W.Z. reviewed and edited the manuscript. Y.Z. researched data and contributed to discussion.

## Supporting information


**Figure S1** Body weight and IPGTT in ApoE^−/−^/LDLR^−/−^ mice.

**Figure S2** The effect of STAMP2 gene overexpression on adipose tissue morphology in ApoE^−/−^/LDLR^−/−^ mice.

**Table S1** Metabolic parameters of ApoE^−/−^LDLR^−/−^ mice
