# Supplementary figures and images for: Overexpressing STAMP2 attenuates adipose tissue angiogenesis and insulin resistance in diabetic ApoE−/−/LDLR −/− mouse via a PPARγ/CD36 pathway
Source: J Cell Mol Med. 2017 Jun 19;21(12):3298–308. doi: 10.1111/jcmm.13233 (PMC5706521; doi:10.1111/jcmm.13233)

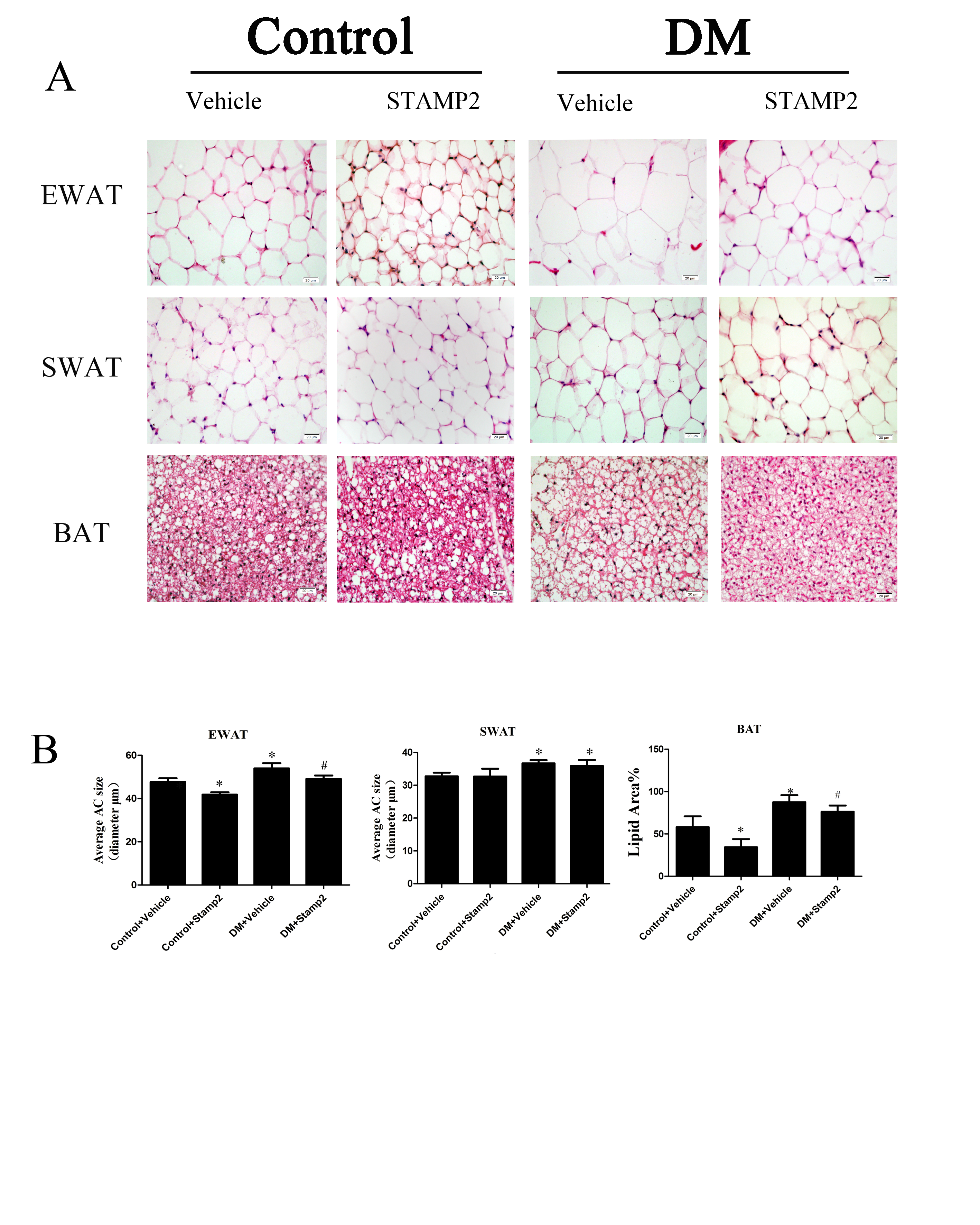

Supplement: Supplementary file 2 — Figure S2 The effect of STAMP2 gene overexpression on adipose tissue morphology in ApoE−/−/LDLR−/− mice. [file JCMM-21-3298-s002.tif]
