# Supplementary material for: Overexpressing STAMP2 attenuates adipose tissue angiogenesis and insulin resistance in diabetic ApoE−/−/LDLR −/− mouse via a PPARγ/CD36 pathway
Source: J Cell Mol Med. 2017 Jun 19;21(12):3298–308. doi: 10.1111/jcmm.13233 (PMC5706521; doi:10.1111/jcmm.13233)
Supplement: Supplementary file 3 — Table S1 Metabolic parameters of ApoE−/−LDLR−/− mice [file JCMM-21-3298-s003.doc]

| **Table S1 Metabolic parameters of ApoE-/-LDLR-/-mice** | | | | |
| --- | --- | --- | --- | --- |
|  | Control+Vehicle (n=6) | Control+STAMP2 (n=10) | DM+Vehicle (n=6) | DM+STAMP2 (n=10) |
| TC | 17.45±1.85 | 19.26±3.05 | 38.42±18.51* | 22.33±5.57^#^ |
| TG | 1.63±0.38 | 1.48±0.42 | 2.38±1.57 | 1.78±0.84 |
| FBG | 6.99±2.44 | 5.59±1.71 | 16.95±11.48* | 8.05±2.4^#^ |
| FFA | 56.2±26.76 | 72.07±30.51 | 96.88±65.37 | 42±16.25^#^ |

Data are expressed as mean±SD. **p*＜0.05 vs. Control+Vehicle; *^#^p*<0.05 vs. DM+Vehicle. Serum sampling was taken under fasting condition and measured. FBG: fasting blood glucose; TG: total triglycerides; TC: total cholesterol; FFA: free fatty acids.
